# What is the additional value of MRI of the foot to the hand in undifferentiated arthritis to predict rheumatoid arthritis development?

**DOI:** 10.1186/s13075-019-1845-7

**Published:** 2019-02-14

**Authors:** Y. J. Dakkak, D. M. Boeters, A. C. Boer, M. Reijnierse, A. H. M. van der Helm-van Mil

**Affiliations:** 1 Department of Rheumatology, Leiden University Medical Centre, P.O. Box 9600, 2300 RC Leiden, The Netherlands; 2 Department of Radiology, Leiden University Medical Centre, Leiden, The Netherlands

**Keywords:** Rheumatoid arthritis, Magnetic resonance imaging, MTP joints

## Abstract

**Background:**

MRI-detected subclinical joint inflammation in the hand joints of patients with undifferentiated arthritis (UA) predicts progression to rheumatoid arthritis (RA). It is unknown if adding MRI of the foot increases predictive accuracy compared to the hand alone.

**Methods:**

1.5-T contrast-enhanced MRI of the unilateral foot (MTP-1-5) and hand (MCP-2-5 and wrist) was performed in 123 patients presenting with UA (not fulfilling the 2010 RA criteria) and scored for bone marrow edema (BME), synovitis and tenosynovitis. Symptom-free controls (*n* = 193) served as a reference for defining an abnormal MRI. Patients were followed for RA development ≤ 1 year, defined as fulfilling the classification criteria or initiation of disease-modifying antirheumatic drugs because of the expert opinion of RA. The added predictive value of foot MRI to hand MRI was evaluated.

**Results:**

Fifty-two percent developed RA. Foot tenosynovitis was predictive (OR 2.55, 95% CI 1.01–6.43), independent of BME and synovitis (OR 3.29, 95% CI 1.03–10.53), but not independent of CRP and number of swollen joints (OR 2.14, 95% CI 0.77–5.95). Hand tenosynovitis was also predictive independent of BME and synovitis (OR 3.99, 95% CI 1.64–9.69) and independent of CRP and swollen joints (OR 2.36, 95% CI 1.04–5.38). Adding foot tenosynovitis to hand tenosynovitis changed the sensitivity from 72 to 73%, specificity from 59 to 54% and AUC from 0.66 to 0.64; the net reclassification index was − 3.5.

**Conclusion:**

MRI-detected tenosynovitis of the foot predicts progression to RA. However, adding MRI of the foot does not improve the predictive accuracy compared to MRI of the hand alone. In view of cost reduction, the performance of foot MRI for prognostic purposes in UA can be omitted.

**Electronic supplementary material:**

The online version of this article (10.1186/s13075-019-1845-7) contains supplementary material, which is available to authorized users.

## Key messages

- MRI-detected tenosynovitis at the MTP joints in patients with undifferentiated arthritis is associated with RA development within 1 year
- Adding information of MRI-detected tenosynovitis at the MTP joints to tenosynovitis of the hand does not increase the predictive accuracy for RA development
- Foot MRI has no additional value to hand MRI in the early detection of RA

## Introduction

In patients with rheumatoid arthritis (RA), early initiation of treatment with disease-modifying antirheumatic drugs (DMARDs) is associated with a better disease outcome [1, 2]. The 2010 ACR/EULAR classification criteria have been developed to facilitate early classification of RA [3]. These criteria perform better in the early identification of anti-citrullinated protein antibody (ACPA)-positive RA than in ACPA-negative RA [4]. This is explained by the fact that three of the six points required to fulfil the criteria can come from autoantibodies and that in the absence of autoantibodies, patients require > 10 involved joints to be classified as RA [5]. Previously, it may have been considered more important to identify ACPA-positive RA early, because of its association with a more destructive disease course. However, with the current treatment strategies, the disease burden and functional status of ACPA-negative patients are similar to that of ACPA-positive patients [6], underlining the equal relevance of early recognition of ACPA-negative RA.

Thus, additional (bio)markers are needed to facilitate the early detection of RA and ACPA-negative patients in particular. Imaging is one of the tools that is being explored. Magnetic resonance imaging (MRI) is increasingly used in clinical trials in RA, as it is a sensitive tool to visualise inflammation as defined by synovitis, bone marrow edema (BME) and tenosynovitis [7]. The use of MRI in undifferentiated arthritis (UA) to predict the progression to clinical RA is recommended by EULAR [8], a recommendation that is supported by the findings of several studies in patients with UA that had RA development as an outcome [9, 10]. Advantages of MRI are its sensitivity and reproducibility; on the other hand, MRI is costly and time-consuming. In order to achieve evidence-based and cost-effective use of MRI in the early detection of RA among patients presenting with UA, the optimal scan protocol needs to be examined. An important issue in this respect is whether imaging of the foot is of additional value to the hand, as it prolongs the scan time and thereby costs [11].

Two studies have performed MRI of the metatarsophalangeal (MTP) joints in UA patients, in addition to MRI of the wrists and metacarpophalangeal (MCP) joints [12, 13]. One study evaluated only BME of the MTP joints (not synovitis and tenosynovitis) [12]. The second study combined data of the hand and foot but did not compare the predictive effect of the hand and foot separately [13]. In addition, both studies did not evaluate tenosynovitis in the foot, whilst in the hand, tenosynovitis has shown to be the most predictive feature of inflammation by MRI [13]. Consequently, the value of MRI-detected inflammation in the foot (synovitis, tenosynovitis or BME) in recognising UA patients that will develop RA is still unknown. With the ultimate aim to arrive at an optimal protocol for MRI in UA, we aimed to assess whether MRI-detected inflammation of the foot is predictive for RA development and whether combining MRI-detected inflammation of the foot to that of the hand is of additional value.

## Methods

### Patients

#### Early arthritis cohort

**Fig. 1:**
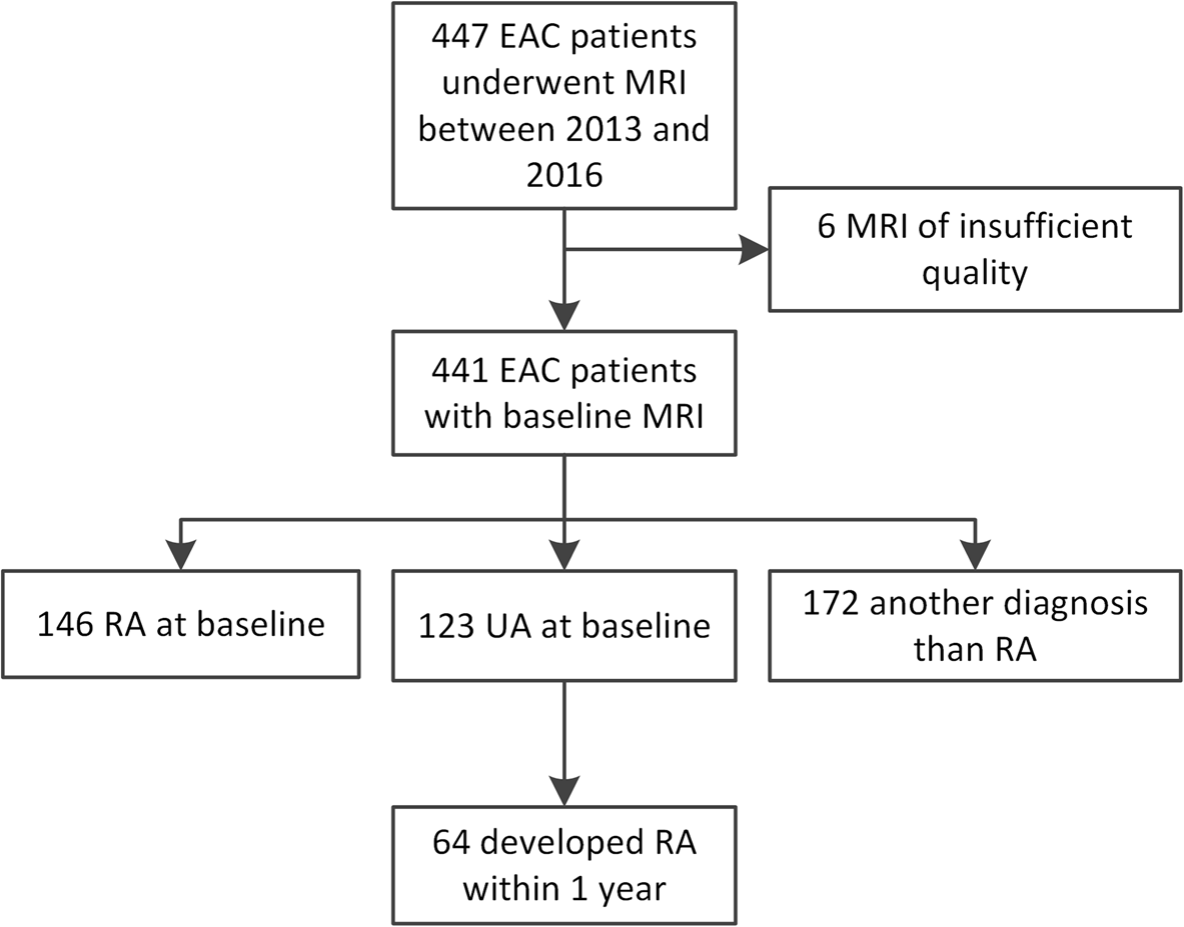
Flowchart. EAC, early arthritic cohort; MRI, magnetic resonance imaging; RA, rheumatoid arthritis; UA, undifferentiated arthritis

The Leiden Early Arthritic Clinic (EAC) is a longitudinal inception cohort that includes patients with clinically confirmed arthritis of ≥ 1 joint and symptom duration of < 2 years that are naïve to DMARDs. At baseline, questionnaires were completed, swollen joint counts were performed and serum samples were obtained. MRI scans were made at baseline, prior to the initiation of DMARDs, and were read later in time for research purposes only; the results were not reported to patients or clinicians. At a second visit, 2 weeks after presentation, patients received their initial diagnosis. UA patients did not fulfil the 2010 criteria and did not receive a diagnosis other than UA at the 2 weeks visit. Between June 2013 and March 2016, 447 consecutive patients presenting with early arthritis were included and underwent MRI. Of these patients, 123 were diagnosed with UA and were studied here (Fig. 1).

#### Symptom-free volunteers

Previously, it was shown that the predictive accuracy of MRI increased and the rate of false-positive findings decreased, when features of low-grade inflammation as observed in the general population were used as reference [14]. Therefore, data from symptom-free controls (*n* = 193) as described before were used as reference [15]. In short, the volunteers were recruited via advertisements in local newspapers and websites and had no history of inflammatory rheumatic diseases, no joint symptoms during the last month and no evidence of arthritis at physical examination [14]. Data on synovitis and BME in the MTP joints were reported previously [15]. For our current study, the presence of tenosynovitis of the flexor and extensor tendons at the level of the MTP joints was also evaluated.

The study was approved by the local medical ethics committee. All participants signed an informed consent.

### MRI and scoring

Patients and symptom-free controls were scanned using the same protocol and scanner, a 1.5-T extremity MRI (General Electric, WI, USA). Unilateral scans were acquired of the MCP joints (2–5), wrist and MTP joints (1–5) of the most painful side or the dominant side, in case of equally severe symptoms and in the symptom-free volunteers. Gd-chelate contrast agent (gadoteric acid (Gd), Guerbet, Paris, France) was administered intravenously at a standard dose of 0.1 mmol/kg. Sequences acquired were coronal pre-contrast T1-weighted fast spin echo (T1), coronal and axial post-contrast T1-weighted fast spin echo with frequency-selective fat saturation (T1Gd) of the MCP joints and wrist and T1Gd in the coronal and axial plane of the MTP joints (Additional file 1). All scanned joints were scored for BME and synovitis according to the Outcome Measures in Rheumatology Clinical Trials Rheumatoid Arthritis Magnetic Resonance Imaging Scoring (OMERACT-RAMRIS) [16]. Tenosynovitis was scored according to Haavardsholm’s score [17] (Additional file 1). Each MRI was scored by two experienced readers, blinded to any clinical data. Intraclass correlation coefficients (ICCs) for the total inflammation scores were calculated to determine the reliability of the readers. All inter- and intrareader ICCs were ≥ 0.93.

### Outcome

The outcome was the development of RA during 1 year follow-up. RA was defined as fulfilling the 2010 criteria and/or the initiation of DMARDs because of the clinical diagnosis of RA that represents the expert opinion of rheumatologists. The fulfilment of the 2010 criteria was not deemed necessary in the presence of a clinical diagnosis and start of DMARDs, as seronegative patients require > 10 involved joints to fulfil the 2010 criteria; in case of a suspected (imminent) RA, patients were generally treated and progression towards acquiring > 10 inflamed joints was hampered.

### Analyses

For continuous scores, the mean MRI scores (BME, synovitis, tenosynovitis) of both readers were used. Next, the MRI scores were dichotomized by using the MRI findings of symptom-free persons as a reference. At joint level, a score was considered abnormal for BME, synovitis or tenosynovitis if the scores of both readers at the same location were present in < 5% of symptom-free persons of the same age category (18–40, 40–60 or > 60 years), as described previously [14]. At patient level, BME, synovitis and tenosynovitis were considered present if ≥ 1 location was positive. The scores of MTP 1–5 were summed as ‘foot’, and MCP 2–5 and the wrist were summed together as ‘hand’.

Tenosynovitis data of the foot of symptom-free persons have not been studied before, in contrast to synovitis and BME [15]. Therefore, we studied this in the symptom-free controls before analysing the data of UA patients.

Continuous MRI data were compared between UA patients with and without progression to RA with Mann-Whitney *U* tests. Logistic regression analysis was used to assess the predictive value of MRI-detected inflammation in UA patients. Multivariable analyses corrected for MRI inflammation features and regularly used inflammatory markers (C-reactive protein (CRP) and swollen joint count). Test characteristics for the development of RA were calculated for the MRI features, and the net reclassification index (MRI of the hand and foot combined versus hand alone) was determined.

Subanalyses were performed with the initiation of DMARDs within 1 year as the outcome and thus not the development of RA according to the classification criteria, next within the subgroup of UA patients who were negative for rheumatoid factor (RF) and ACPA (*n* = 110), with data for the flexor and extensor tendons of both the hand and foot separately and with data for the MCP joints and wrist separately (rather than summed together as the hand).

*P* values less than 0.05 were considered significant. IBM SPSS v23.0 was used.

## Results

### Study population

Baseline characteristics are presented in Table 1. UA patients had a mean age of 57, 59% were female, and they were mainly autoantibody-negative (95%). After 1 year follow-up, 64 patients (52%) had developed RA (6 received DMARDs and fulfilled the classification criteria, 57 received DMARDs but did not fulfil the 2010 criteria and 1 fulfilled the criteria but did not receive DMARDs). Patients who developed RA had more swollen joints and a higher CRP as is presented in Table 1.

**Table 1:** Baseline characteristics of all undifferentiated arthritis patients and those who progress to RA after 1 year and those who do not

	UA at baseline, *N* = 123	RA after 1 year, *N* = 64	No RA after 1 year, *N* = 59	*P* value
Clinical parameters				
Age, mean (SD)	57 (18)	59 (19)	55 (16)	0.29
Female, *n* (%)	73 (59)	40 (63)	33 (56)	0.46
Symptom duration, in weeks, median (IQR)	9 (4–27)	12 (6–30)	8 (3–21)	0.048
Swollen joint count, median (IQR)	2 (1–4)	3 (2–5)	1 (1–3)	< 0.001
Presence of swollen joint foot, *n* (%)	25 (20)	12 (20)	13 (22)	0.65
Presence of swollen joint hand, *n* (%)	66 (54)	40 (63)	26 (44)	0.041
Presence of tender joint foot, *n* (%)	32 (26)	17 (27)	15 (25)	0.89
Presence of tender joint hand, *n* (%)	71 (58)	44 (69)	27 (46)	0.010
CRP, mg/L, median (IQR)	5 (3–14)	6 (3–16)	3 (3–9)	0.035
ACPA positive, *n* (%)	6 (5)	4 (7)	2 (3)	0.44
RF positive, *n* (%)	8 (7)	5 (8)	3 (5)	0.53
MRI features				
MTP 1–5				
Tenosynovitis, mean (SD)	0.6 (1.3)	0.8 (1.4)	0.4 (1.1)	0.075
Synovitis, mean (SD)	0.7 (1.0)	0.7 (1.0)	0.7 (1.1)	0.69
BME, mean (SD)	0.8 (1.4)	0.6 (0.9)	1.0 (1.7)	0.50
MCP 2–5				
Tenosynovitis, mean (SD)	1.4 (2.0)	2.0 (2.3)	0.8 (1.2)	< 0.001
Synovitis, mean (SD)	1.6 (1.8)	2.1 (1.9)	1.1 (1.5)	0.001
BME, mean (SD)	0.6 (1.5)	0.6 (1.3)	0.7 (1.6)	0.85
Wrist				
Tenosynovitis, mean (SD)	2.4 (3.1)	3.2 (3.5)	1.5 (2.4)	0.006
Synovitis, mean (SD)	1.9 (2.1)	2.4 (2.3)	1.5 (1.8)	0.023
BME, mean (SD)	3.0 (5.2)	3.5 (5.8)	2.5 (4.5)	0.57

Sixty-six swollen joint counts were performed. For the presence of a swollen or tender foot, the presence of a swollen or tender MTP joint was taken, respectively. For the presence of swollen or tender hand, the presence of a swollen or tender MCP or wrist joint was taken, respectively. Differences between patients with and without progression to RA after 1 year were tested with chi-square test, *t* test or Mann-Whitney *U* test as appropriate

*UA* undifferentiated arthritis, *SD* standard deviation, *IQR* interquartile range, *CRP* C-reactive protein, *RF* rheumatoid factor, *ACPA* anti-citrullinated protein antibody, *MCP* metacarpophalangeal joint, *MTP* metatarsophalangeal joint, *BME* bone marrow edema

### Continuous MRI scores

Continuous MRI scores are presented in Table 1. For the foot BME and synovitis, the scores were comparable in UA patients who did and did not develop RA (*P* = 0.50 and *P* = 0.69, respectively); tenosynovitis scores were higher in patients that progressed to RA (mean score 0.8 versus 0.4), although the difference did not reach statistical significance (*P* = 0.075).

### Frequency of tenosynovitis at the MTP joints in symptom-free controls

Studying the frequency of tenosynovitis at the MTP joints in symptom-free persons for different age categories revealed that tenosynovitis never occurred in > 5% of patients (Table 2). Thus, the presence of tenosynovitis at the foot was always considered abnormal.

**Table 2:** Frequencies of flexor and extensor tenosynovitis scores at the MTP joints of 193 healthy controls

	Tenosynovitis score			
	0	1	2	3
Flexor tenosynovitis				
MTP 1	192 (99.5%)	1 (0.5%)	–	–
MTP 2	192 (99.5%)	1 (0.5%)	–	–
MTP 3	192 (99.5%)	1 (0.5%)	–	–
MTP 4	193 (100%)	–	–	–
MTP 5	193 (100%)	–	–	–
Extensor tenosynovitis				
MTP 1	193 (100%)	–	–	–
MTP 2	193 (100%)	–	–	–
MTP 3	193 (100%)	–	–	–
MTP 4	192 (99.5%)	1 (0.5%)	–	–
MTP 5	193 (100%)	–	–	–

A dash (–) indicates absence in healthy controls (0%)

*MTP* metatarsophalangeal joint

### Prediction of RA development within 1 year for UA patients

Then MRI features of UA patients were dichotomized according to a cutoff based on the findings in symptom-free controls as described in the “Methods” section and are done previously [14]. Results of univariable logistic regression are depicted in Table 3. Of all MRI features of the foot, only tenosynovitis was associated with RA development, as it occurred in 29% of patients that progressed to RA and in 14% of those that did not (OR 2.55, 95% CI 1.01–6.43). Tenosynovitis regularly co-occurred with synovitis and BME, as is illustrated in Additional file 1: Figure S1. Therefore, a multivariable analysis was performed for all three types of inflammation of the foot. Tenosynovitis predicted the outcome RA independently of local BME and synovitis of the foot (OR 3.29, 95% CI 1.03–10.53). In a multivariable analysis that adjusted tenosynovitis for CRP and swollen joint count, the OR for tenosynovitis was 2.14 (95% CI 0.77–5.95).

**Table 3:** Results of logistic regression for RA development in undifferentiated arthritis patients

	Patients with MRI feature, *n* (%)		Univariable analyses		Multivariable analyses: types of MRI inflammation^b^		Multivariable analysis: presence of tenosynovitis adjusted for SJC and CRP^b^	
	RA	No RA	OR (95% CI)	*P* value	OR (95% CI)	*P* value	OR (95% CI)	*P* value
Foot (MTPs)^a^								
Presence of:								
Tenosynovitis	18 (29)	8 (14)	2.55 (1.01–6.43)	0.047	3.29 (1.03–10.53)	0.045	2.14 (0.77–5.95)	0.14
Synovitis	14 (22)	10 (17)	1.40 (0.57–3.45)	0.47	0.96 (0.30–3.10)	0.95		
BME	6 (9)	11 (19)	0.44 (0.16–1.31)	0.14	0.33 (0.10–1.09)	0.068		
Hand (MCPs and wrist)^a^								
Presence of:								
Tenosynovitis	46 (72)	24 (41)	3.73 (1.76–7.91)	0.001	3.99 (1.64–9.69)	0.002	2.36 (1.04–5.38)	0.041
Synovitis	28 (44)	18 (31)	1.77 (0.84–3.72)	0.13	1.04 (0.41–2.67)	0.94		
BME	21 (33)	24 (41)	0.71 (0.34–1.49)	0.37	0.57 (0.25–1.29)	0.18		
Swollen joints, per joint			1.46 (1.20–1.79)	< 0.001				
Elevated CRP			2.77 (1.23–6.23)	0.014				

*SJC* swollen joint count (66 swollen joint counts were performed), *CRP* C-reactive protein, *RA* rheumatoid arthritis, *OR* odds ratio, *CI* confidence interval, *MTP* metatarsophalangeal joint, *MCP* metacarpophalangeal joint, *BME* bone marrow edema

^a^At joint level, a score was considered abnormal for BME, synovitis or tenosynovitis if the scores of both readers at the same location were present in < 5% of symptom-free persons of the same age category (18–40, 40–60 or > 60 years). At patient level, BME, synovitis and tenosynovitis were considered present if ≥ 1 joint of foot or hand was positive

^b^Multivariable analyses were performed for the foot and for the hand separately

**Fig. 2:**
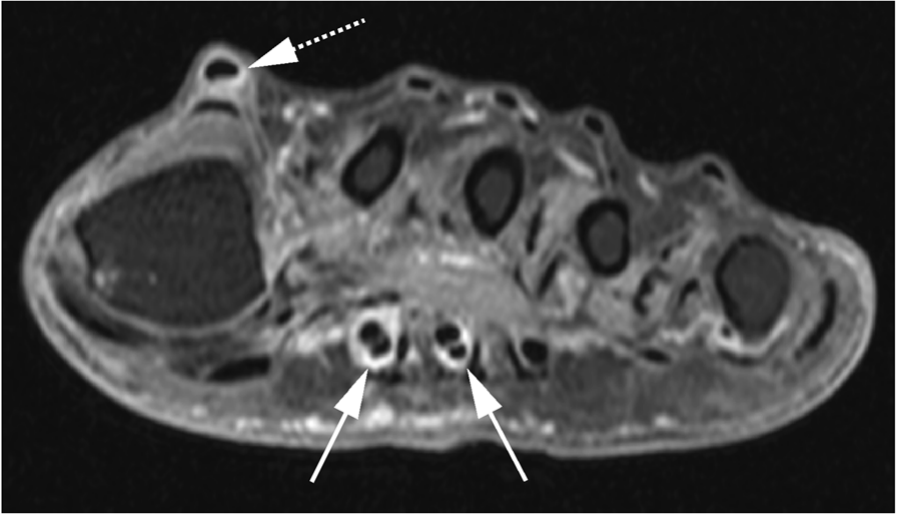
Example of tenosynovitis in patient with undifferentiated arthritis who developed RA. The axial image portrays tenosynovitis at the flexor of metatarsophalangeal (MTP)-2 and MTP-3 (arrows with line) and the extensor of MTP-1 (arrow with dotted line)

The test characteristics of tenosynovitis of the foot to identify RA patients were as follows: sensitivity 29%, specificity 86%, positive predictive value (PPV) 69%, negative predictive value (NPV) 53% and area under the curve (AUC) 0.58 (Table 4). An example of MRI-detected tenosynovitis of the foot is shown in Fig. 2.

**Table 4:** Test characteristics of the MRI features in patients with UA for RA development

	Sensitivity	Specificity	PPV	NPV	Accuracy	AUC
Foot (MTPs)						
Tenosynovitis	29 (19–41)	86 (75–93)	69 (50–83)	53 (43–63)	57 (48–65)	0.58
Synovitis	22 (14–34)	83 (72–91)	58 (39–76)	50 (40–60)	52 (43–60)	0.53
BME	9 (4–19)	81 (70–89)	35 (17–59)	45 (36–55)	44 (35–53)	0.45
Any Inflammation	38 (27–50)	68 (55–78)	56 (41–70)	50 (39–61)	52 (43–61)	0.53
Hand (MCPs and wrist)						
Tenosynovitis	72 (60–81)	59 (47–71)	66 (54–76)	66 (53–77)	66 (57–74)	0.66
Synovitis	44 (32–56)	69 (57–80)	61 (46–74)	53 (42–64)	56 (47–65)	0.57
BME	33 (23–45)	59 (47–71)	47 (33–61)	45 (34–56)	46 (37–54)	0.46
Any Inflammation	77 (65–85)	36 (25–48)	56 (46–66)	58 (42–65)	57 (48–65)	0.56
Hand or foot						
Tenosynovitis	73 (62–83)	54 (42–66)	64 (52–74)	65 (51–77)	64 (55–72)	0.64
Synovitis	48 (37–60)	59 (47–71)	56 (43–69)	51 (40–63)	54 (45–62)	0.54
BME	39 (28–51)	47 (35–60)	45 (32–58)	42 (31–54)	43 (35–52)	0.43
Any Inflammation	78 (67–86)	24 (15–36)	53 (43–62)	50 (33–67)	52 (43–61)	0.51

Any Inflammation is defined as the presence of BME, synovitis and/or tenosynovitis. Values are depicted with their corresponding 95% confidence intervals between brackets

*PPV* positive predictive value, *NPV* negative predictive value, *AUC* area under the curve, *MTP* metatarsophalangeal joint, *MCP* metacarpophalangeal joint, *BME* bone marrow edema

### Additional value of MRI of the foot over the hand

Since at the foot level the association of an abnormal MRI with the development of RA was the strongest for tenosynovitis, further analyses were confined to tenosynovitis. To answer the question whether imaging of the foot is of additional value to imaging the hand, first, the value of MRI of the hand was assessed. Of all MRI features of the hand, tenosynovitis was the only predictor for the outcome (OR 3.73, 95% CI 1.76–7.91), as was previously shown [13]. This effect was independent of local BME and synovitis (OR 3.99, 95% CI 1.64–9.69) and also independent of clinical inflammatory markers (OR 2.36, 95% CI 1.04–5.38) (Table 3).

Tenosynovitis of the hand had a sensitivity of 72%, specificity of 59%, PPV of 66%, NPV of 66% and AUC of 0.66 for the identification of RA patients, as is presented in Table 4. To evaluate if scoring tenosynovitis of the foot is of additional value, the test characteristics of the hand and foot combined were calculated. This resulted in a comparable sensitivity of 72% and 73%, at the cost of a decrease in specificity from 59 to 54% and a change in AUC from 0.66 to 0.64 (Table 4). The net reclassification index was − 3.5 (as is illustrated in Additional file 1: Table S1).

### Subanalyses

We repeated the analyses for the association between tenosynovitis at the foot for UA patients who received DMARDs within 1 year, although they did not fulfil the 2010 classification criteria (*n* = 57). The OR for the association of tenosynovitis was 2.76 (95% CI 1.12–6.82) in univariable analyses and 2.96 (95% CI 0.97–9.01) in multivariable analyses corrected for local BME and synovitis (Additional file 1: Table S2).

The association between tenosynovitis at the foot and RA development was also studied in the UA patients who were negative for RF and ACPA (*n* = 110), as it has been suggested that MRI may be most helpful in the ACPA-negative subgroup [13]. Then, the OR of tenosynovitis at the MTP joints was 2.06 (95% CI 0.78–5.40) in univariable analyses and 2.62 (95% CI 0.79–8.68) when corrected for local BME and synovitis (Additional file 1: Table S3).

In the main analyses, inflammation at the flexor and at the extensor tendons was summed. It was also assessed whether the predictive value of foot tenosynovitis differed between the two sides (Additional file 1: Table S4). The effect remained present for the flexor side (22% versus 9% of patients, OR 3.09, 95% CI 1.04–9.20) but not for the extensor side (14% versus 9% of patients, OR 1.80, 95% CI 0.57–5.72).

It was observed that tenosynovitis at the hand level had a larger effect size (OR 3.73, 95% CI 1.76–7.91) than that at the foot (2.55, 95% CI 1.01–6.43) (Table 1). Thus far, in the analyses of the hand, tenosynovitis of the wrist and MCP joints was summed. To explore whether the fact that more tendons were scored in the hand (yielding a higher chance of MRI positivity), explained this finding, we repeated the analyses for the MCP joints and wrist separately and compared the MCP and MTP joints (Additional file 1: Table S5). The predictive effect of tenosynovitis of the MCP joints remained present (OR 3.64, 95% 1.68–7.91), also after correction for other MRI inflammatory features and after correction for CRP and swollen joints (OR 3.61, 95% CI 1.50–8.65 and OR 2.64, 95% CI 1.14–6.16, respectively). This was in contrast to the results of tenosynovitis of the MTP joints (OR 2.14, 95% CI 0.77–5.95) (Table 3). This suggests that tenosynovitis of the hand joints is truly stronger associated with RA development than that of tenosynovitis of the foot joints.

Finally, we wondered if the lack of an additive effect of foot MRI to hand MRI is based on the concurrent presence of foot tenosynovitis and hand tenosynovitis. Indeed, we observed that 3% of UA patients had foot tenosynovitis without hand tenosynovitis, whereas 60% of UA patients had foot tenosynovitis as well as hand tenosynovitis.

## Discussion

The presence of MRI-detected inflammation has been shown to be predictive for RA development in patients with UA, and the use of MRI in the diagnostic process of RA has been advocated by the EULAR [8, 12, 13]. However, the value of MRI of the foot has not been studied thoroughly. Therefore, we assessed the added benefit of MRI of the foot to MRI of the hand. We observed that of all features of MRI-detected inflammation (BME, synovitis and tenosynovitis) in the foot, tenosynovitis was the only feature to predict progression to RA, consistent with the hand. However, the presence of tenosynovitis in the foot did not have additional value to tenosynovitis in the hand in detecting patients who progressed to RA.

Although MRI-detected tenosynovitis in the foot had a high specificity (86%), and was thus an infrequent occurrence in the group of patients that did not develop RA, it had a low sensitivity (29%). Therefore, scanning the foot instead of the hand is not advisable as the majority of the patients that will progress to RA will remain undetected.

As RA is a disease characterised by inflammation of the small joints of the hands and feet [18], the negative finding on the added value of foot MRI may be unexpected. A presumable explanation is that the foot is less affected in UA. UA is, as shown, a population that is mostly autoantibody negative. The notion that ACPA-negative patients have a lower frequency of foot involvement is supported by a recent finding done at symptom level in patients with arthralgia suspicious for progression to RA; ACPA-negative patients less often had symptoms in the lower extremities than ACPA-positive patients [19]. In the UA patients studied here, the frequency of foot involvement at joint examination was lower than hand involvement (lower number of tender and swollen joints) (Table 1), and also, MR-detected tenosynovitis of the feet was less frequent than in the hand (21% versus 57% on group level). More importantly, only 3% of the UA patients had MTP tenosynovitis without concomitant hand tenosynovitis (Additional file 1: Table S1). Thus, although tenosynovitis of the foot was associated with the development of RA, it occured infrequently without hand tenosynovitis, and this may explain why MRI of the foot is not of added benefit.

An absence of added benefit for foot MRI has been observed in different disease phases. MRI-detected erosions of the foot were not of added value to MRI-detected erosions of the hand in identifying RA patients with erosive progression, and in a trial, MRI-detected erosions and synovitis of the foot did not have much additional value to MRI of the hands in evaluating MRI-detected improvement in different treatment arms [20, 21].

This is the first study in UA reporting on the predictive value of tenosynovitis at the MTP joints. The finding in the foot that, of all inflammatory features, tenosynovitis is the most predictive for RA development is in line with the previously published findings on tenosynovitis in the hand [13]. Our study also newly reports on the absence of tenosynovitis in the MTP joints in symptom-free persons from the general population. This highlights the relevance of tenosynovitis as an early phenomenon in RA development [22].

Tenosynovitis was defined as inflammation present at either the flexor or the extensor tendon of the joint, similar as was done in a previous study evaluating tenosynovitis at the level of the MCP joints [23]. For the MCP joints, it is known that the flexor tendons have a tendon sheath, whereas the extensor tendons do not, and inflammation detected around this tendon may better be named peritendinitis [23]. The anatomy with respect to the presence and extent of tendons sheaths at the flexor and extensor tendons at the MTP joints is less clear and defined differently in anatomic atlases, but there seems to be an agreement on the absence of a sheath at the extensor side. Also here, although peritendinitis may be a better term for the signal at the extensor site, for reasons of simplicity, we kept the naming ‘tenosynovitis’ for the signal intensity observed around the tendons at MRI. Nonetheless, the anatomy and the nature of tendon inflammation at the MTP joints are subject of further studies [11].

The development of RA was used as an outcome. This was defined as fulfilling the 2010 criteria and/or as the start of DMARDs by rheumatologists based on their clinical expertise. The latter was considered an outcome without the fulfilment of the 2010 criteria, as these criteria are difficult to fulfil for patients without autoantibodies, as > 10 involved joints are required, and initiated DMARDs may hamper the progression to the fulfilment of RA classification criteria. Indeed in our data, most patients that were treated did not achieve sufficient points over time to fulfil the 2010 criteria. We cannot exclude that this resulted in a frequency of RA development that is higher than would have been observed if patients were not treated and the natural course would have been observed. This could have resulted in overdiagnosis of RA and could have diluted our results leading to an underestimation of the obtained effects for MRI inflammation. However, this probably has not affected the results of the additive value of foot MRI in UA.

Excluding the foot from the MRI protocol saves time. We used a 1.5-T MSK scanner, and deletion of the foot saves 10 min of a total protocol of 45 min. A 3-T MRI is more often used; here, deletion of the foot and scanning according to our protocol would roughly save 10 min from the total time of 30 min, saving a third of the scanning time and thereby reducing costs.

## Conclusion

Patients with UA are mostly autoantibody-negative; in these patients, MRI-detected tenosynovitis of the foot associates with an increased risk to develop RA. However, MRI of the foot does not have additive predictive value to MRI of the hand. This contributes to evidence-based scanning protocols in UA and may reduce costs, as MRI of the foot can be omitted when MRI of the hand is made.

## Additional file

Additional file 1: Supplementary material. (DOCX 92 kb)

## Abbreviations

ACPA: Anti-citrullinated protein antibody
AUC: Area under the curve
BME: Bone marrow edema
CI: Confidence interval
CRP: C-reactive protein
DMARDS: Disease-modifying antirheumatic drugs
EAC: Early Arthritis Clinic
Gd: Gadoteric acid
ICC: Intraclass correlation coefficients
MCP joints: Metacarpophalangeal joints
MRI: Magnetic resonance imaging
MSK: Musculoskeletal
MTP joints: Metatarsophalangeal joints
NPV: Negative predictive value
OMERACT: Outcome Measures in Rheumatology Clinical Trials
OR: Odds ratio
PPV: Positive predictive value
RA: Rheumatoid arthritis
RAMRIS: RA MRI score
RF: Rheumatoid factor
UA: Undifferentiated arthritis

## References

[1] Furst DE, Pangan AL, Harrold LR (2011). Greater likelihood of remission in rheumatoid arthritis patients treated earlier in the disease course: results from the Consortium of Rheumatology Researchers of North America registry. Arthritis Care Res (Hoboken)..

[2] Kyburz D, Gabay C, Michel BA (2011). The long-term impact of early treatment of rheumatoid arthritis on radiographic progression: a population-based cohort study. Rheumatology (Oxford).

[3] Aletaha D, Neogi T, Silman AJ (2010). 2010 Rheumatoid Arthritis Classification Criteria: an American College of Rheumatology/European League Against Rheumatism collaborative initiative. Arthritis Rheum.

[4] Boeters DM, Gaujoux-Viala C, Constantin A (2017). The 2010 ACR/EULAR criteria are not sufficiently accurate in the early identification of autoantibody-negative rheumatoid arthritis: results from the Leiden-EAC and ESPOIR cohorts. Semin Arthritis Rheum.

[5] van der Helm-van Mil AH, Zink A (2017). What is rheumatoid arthritis? Considering consequences of changed classification criteria. Ann Rheum Dis.

[6] Boer AC, Boonen A, van der Helm-van Mil AHM (2018). Is anti-citrullinated protein antibody-positive rheumatoid arthritis still a more severe disease than anti-citrullinated protein antibody-negative rheumatoid arthritis? A longitudinal cohort study in rheumatoid arthritis patients diagnosed from 2000 onward. Arthritis Care Res (Hoboken).

[7] Peterfy C, Ostergaard M, Conaghan PG (2013). MRI comes of age in RA clinical trials. Ann Rheum Dis.

[8] Colebatch AN, Edwards CJ, Ostergaard M (2013). EULAR recommendations for the use of imaging of the joints in the clinical management of rheumatoid arthritis. Ann Rheum Dis.

[9] Eshed I, Feist E, Althoff CE (2009). Tenosynovitis of the flexor tendons of the hand detected by MRI: an early indicator of rheumatoid arthritis. Rheumatology (Oxford).

[10] Machado PM, Koevoets R, Bombardier C (2011). The value of magnetic resonance imaging and ultrasound in undifferentiated arthritis: a systematic review. J Rheumatol Suppl.

[11] Dakkak YJ, van der Heijde DM, Reijnierse M (2018). Validity of the rheumatoid arthritis MRI score applied to the forefeet using the OMERACT filter: a systematic literature review. RMD Open.

[12] Duer-Jensen A, Horslev-Petersen K, Hetland ML (2011). Bone edema on magnetic resonance imaging is an independent predictor of rheumatoid arthritis development in patients with early undifferentiated arthritis. Arthritis Rheum.

[13] Nieuwenhuis WP, van Steenbergen HW, Mangnus L, et al. Evaluation of the diagnostic accuracy of hand and foot MRI for early rheumatoid arthritis. Rheumatology (Oxford). 2017;56(8):1367-77. 10.1093/rheumatology/kex167 28460018

[14] Boer AC, Burgers LE, Mangnus L (2017). Using a reference when defining an abnormal MRI reduces false-positive MRI results-a longitudinal study in two cohorts at risk for rheumatoid arthritis. Rheumatology (Oxford).

[15] Mangnus L, van Steenbergen HW, Reijnierse M (2016). Magnetic resonance imaging-detected features of inflammation and erosions in symptom-free persons from the general population. Arthritis Rheumatol..

[16] Ostergaard M, Peterfy C, Conaghan P (2003). OMERACT rheumatoid arthritis magnetic resonance imaging studies. Core set of MRI acquisitions, joint pathology definitions, and the OMERACT RA-MRI scoring system. J Rheumatol.

[17] Haavardsholm EA, Ostergaard M, Ejbjerg BJ (2007). Introduction of a novel magnetic resonance imaging tenosynovitis score for rheumatoid arthritis: reliability in a multireader longitudinal study. Ann Rheum Dis.

[18] Grondal L, Tengstrand B, Nordmark B (2008). The foot: still the most important reason for walking incapacity in rheumatoid arthritis: distribution of symptomatic joints in 1,000 RA patients. Acta Orthop.

[19] Burgers LE, van Steenbergen HW, Ten Brinck RM (2017). Differences in the symptomatic phase preceding ACPA-positive and ACPA-negative RA: a longitudinal study in arthralgia during progression to clinical arthritis. Ann Rheum Dis.

[20] Durez P, Malghem J, Nzeusseu Toukap A (2007). Treatment of early rheumatoid arthritis: a randomized magnetic resonance imaging study comparing the effects of methotrexate alone, methotrexate in combination with infliximab, and methotrexate in combination with intravenous pulse methylprednisolone. Arthritis Rheum.

[21] Ejbjerg BJ, Vestergaard A, Jacobsen S (2005). The smallest detectable difference and sensitivity to change of magnetic resonance imaging and radiographic scoring of structural joint damage in rheumatoid arthritis finger, wrist, and toe joints: a comparison of the OMERACT rheumatoid arthritis magnetic resonance imaging score applied to different joint combinations and the Sharp/van der Heijde radiographic score. Arthritis Rheum.

[22] Niemantsverdriet E, van der Helm-van Mil AHM (2018). Imaging detected tenosynovitis of metacarpophalangeal and wrist joints: an increasingly recognised characteristic of rheumatoid arthritis. Clin Exp Rheumatol.

[23] Nieuwenhuis WP, Krabben A, Stomp W (2015). Evaluation of magnetic resonance imaging-detected tenosynovitis in the hand and wrist in early arthritis. Arthritis Rheumatol.

