# Supplementary material for: What is the additional value of MRI of the foot to the hand in undifferentiated arthritis to predict rheumatoid arthritis development?
Source: Arthritis Res Ther. 2019 Feb 14;21:56. doi: 10.1186/s13075-019-1845-7 (PMC6376768; doi:10.1186/s13075-019-1845-7)
Supplement: Supplementary file 1 — Supplementary material. (DOCX 92 kb) [file 13075_2019_1845_MOESM1_ESM.docx]

**Additional file 1**

# Detailed MRI protocol

MR imaging was performed on a MSK-extreme 1.5T extremity MR imaging system (GE, Wisconsin, USA) using a 145mm coil for the foot and a 100mm coil for the hand. The patient was positioned in a chair beside the scanner, with the hand or foot fixed in the coil with cushions.

In the hand (MCP2-5 and wrist) the following sequence was acquired before contrast administration: T1-weighted fast spin-echo (FSE) sequence in the coronal plane (repetition time (TR) 575 ms, echo time (TE) 11.2 ms, acquisition matrix 388×288, echo train length (ETL) 2). After intravenous injection of gadolinium contrast (gadoteric acid, Guerbet, Paris, France, standard dose of 0.1 mmol/kg) the following sequences were obtained: T1-weighted FSE sequence with frequency selective fat saturation (fatsat) in the coronal plane (TR/TE 700/9.7ms, acquisition matrix 364×224, ETL 2), T1-weighted FSE sequence with frequency selective fat saturation in the axial plane (wrist: TR/TE 540/7.7 ms; acquisition matrix 320x192; ETL 2 and MCP-joints: TR/TE 570/7.7 ms; acquisition matrix 320x192; ETL 2).

The following sequences of the forefoot (MTP1-5) were obtained after intravenous injection of gadolinium contrast: T1-weighted FSE fatsat sequence in the axial plane (TR/TE 700/9.5ms; acquisition matrix 364x224, ETL 2) and: T1-weighted FSE fatsat sequence in the coronal plane (perpendicular to the axis of the MTPs) (TR/TE 540/7.5ms; acquisition matrix 320x192, ETL 2). Field-of-view was 100mm for the hand and 140mm for the foot. Coronal sequences of the hand had 18 slices with a slice thickness of 2mm and a slice gap of 0.2mm. Coronal sequences of the foot had 20 slices with a slice thickness of 3mm and a slice gap of 0.3mm. All axial sequences had a slice thickness of 3mm and a slice gap of 0.3mm with 20 slices for the wrist, 16 for the metacarpophalangeal-joints and 14 for the foot.

According to the RAMRIS-method, T2-weighted fat suppressed or short tau inversion recovery (STIR) sequences should be used to assess bone marrow edema (BME). Previously, three studies have demonstrated that a contrast enhanced T1-weigthed fat suppressed sequence has a strong correlation with T2-weighted fat suppressed sequences.(1-3) The European Society of Musculoskeletal Radiology (ESSR) Arthritis Subcommittee also recommends the use of contrast enhanced T1-weighted fat suppressed sequences for depiction of BME.(4) We used the contrast enhanced T1-weighted fat suppressed sequence as it allowed a shorter scan time and has a higher signal to noise ratio.

# MRI scoring

The metatarsophalangeal(MTP)-, metacarpophalangeal(MCP)- and wrist- bones and joints were scored in line with the validated RA MRI scoring system (RAMRIS).(5) For tenosynovitis, the score as described by Havaardsholm was applied to the wrist and in addition to the extensor and flexor tendons of the MTPs and MCPs.(6) Erosions were scored on a 0-10 scale based on the percentage of eroded bone (0, no eroded bone, 1 >0-10%, 2 11-20% etc.), Bone marrow edema (BME) was scored on a 0-3 scale based on the affected volume of the bone (no BME, >0-33%, >33-66%, >66%), the synovitis score (range 0-3) was scored based on the volume of enhancing tissue in the synovial compartment (none, mild, moderate, severe) and the tenosynovitis-score (ranged 0-3) was based on the thickness of peritendinous effusion or synovial proliferation with contrast enhancement (normal, <2mm, 2-5mm, >5mm).

The scores of proximal and distal BME were summed into BME scores and scores of flexor and extensor tenosynovitis into tenosynovitis scores. For the foot the scores of MTP1-5 were summed, in the hand the scores of MCP 2-5 and of the wrist bones and tendons were summed.

Missing scores: Infrequently erosions, BME, synovitis or tenosynovitis could not be reliably assessed. This was mostly due to inhomogeneous fat suppression or movement artifacts. In total, 1.0% of MRI scores were missing. This was considered to be completely at random and was not imputed.

# Table S1: Reclassification of patients by adding MRI-detected tenosynovitis of the foot to that of the hand with RA-development after one year as an outcome

|  | RA within 1 year | |  |
| --- | --- | --- | --- |
|  | no RA | RA | Total |
| MRI of hand |  |  |  |
| - No tenosynovitis | 35 | 18 | 53 |
| - Tenosynovitis | **24** | **46** | 70 |
| MRI of hand and foot |  |  |  |
| - No tenosynovitis in hand or foot | 32 | 17 | 49 |
| - Tenosynovitis in hand or foot | **27** | **47** | 74 |
| Total | 59 | 64 | 123 |

The hand is defined as MCP 2-5 or the wrist, the foot is defined as MTP 1-5.

By adding MRI-detected tenosynovitis of the feet to that of the hand the net proportion of reclassifications was 3.3% ((3+1)/123).

The net increase in correct classifications was 1.6% (1/64), the net increase in incorrect classifications was 5.1% (3/59). Thus the net reclassification index was **-3.5** (1.6-5.1).

# Table S2: Results of logistic regression with initiation of DMARDs as the outcome in undifferentiated arthritis patients

|  | **Patients with MRI-feature, n (%)** | | **Univariable analyses** | | **Multivariable analyses: types of MRI-inflammation²** | | **Multivariable analysis:**  **presence of tenosynovitis adjusted for SJC and CRP²** | |
| --- | --- | --- | --- | --- | --- | --- | --- | --- |
|  | RA | no-RA | OR (95% CI) | P-value | OR (95% CI) | P-value | OR (95% CI) | P-value |
|  |  |  |  |  |  |  |  |  |
| **Foot (MTPs)¹**  Presence of | |  |  |  |  |  |  |  |
| Tenosynovitis | 17 (30) | 9 (14) | 2.76 (1.12-6.82) | 0.028 | 2.96 (0.97-9.01) | 0.056 | 2.28 (0.81-6.42) | 0.12 |
| Synovitis | 14 (25) | 10 (15) | 1.87 (0.76-4.61) | 0.18 | 1.47 (0.46-4.67) | 0.52 |  |  |
| BME | 5 (9) | 12 (18) | 0.43 (0.14-1.31) | 0.14 | 0.27 (0.08-0.97) | 0.045 |  |  |
| **Hand (MCPs and wrist)¹**  Presence of | | |  |  |  |  |  |  |
| Tenosynovitis | 44 (77) | 26 (39) | 5.21 (2.36-11.49) | <0.001 | 5.24 (2.10-13.09) | <0.001 | 3.33 (1.39-7.97) | 0.007 |
| Synovitis | 27 (47) | 19 (29) | 2.23 (1.06-4.69) | 0.035 | 1.19 (0.46-3.04) | 0.72 |  |  |
| BME | 19 (33) | 26 (39) | 0.77 (0.37-1.61) | 0.49 | 0.57 (0.24-1.32) | 0.19 |  |  |
| Swollen joints,  per joint | | | 1.47 (1.21-1.78) | <0.001 |  |  |  |  |
| Elevated CRP | |  | 3.96 (1.74-9.00) | 0.001 |  |  |  |  |

¹At joint level a score was considered abnormal for BME, synovitis or tenosynovitis if the scores of both readers at the same location was present in <5% of symptom-free persons of the same age category (18-40, 40-60, or >60 years). At patient level, BME, synovitis, tenosynovitis were considered present if ≥1 joint of foot or hand respectively was positive. ²Multivariable analyses was performed for the foot and for the hand separately. DMARD: disease modifying antirheumatic drug; SJC: swollen joint count (66 swollen joint counts were performed); CRP: C-reactive protein; OR: odds ratio; CI: confidence interval; MTP: metatarsophalangeal joint; MCP: metacarpophalangeal joint; BME: bone marrow edema.

# Table S3: Results of logistic regression for RA development in undifferentiated arthritis patients who are antibody negative

|  | **Patients with MRI-feature, n (%)** | | **Univariable analyses** | | **Multivariable analyses: types of MRI-inflammation²** | | **Multivariable analysis: presence of tenosynovitis, adjusted for SJC and CRP²** | |
| --- | --- | --- | --- | --- | --- | --- | --- | --- |
|  | RA | no-RA | OR (95% CI) | P-value | OR (95% CI) | P-value | OR (95% CI) | P-value |
|  |  |  |  |  |  |  |  |  |
| **Foot (MTPs)¹** | |  |  |  |  |  |  |  |
| Tenosynovitis | 14 (26) | 8 (15) | 2.06 (0.78-5.40) | 0.14 | 2.62 (0.79-8.68) | 0.12 | 1.89 (0.64-5.56) | 0.25 |
| Synovitis | 11 (20) | 9 (16) | 1.31 (0.49-3.46) | 0.59 | 1.19 (0.34-4.16) | 0.79 |  |  |
| BME | 5 (9) | 11 (20) | 0.40 (0.13-1.24) | 0.11 | 0.27 (0.075-1.01) | 0.051 |  |  |
| **Hand (MCPs and wrist)¹** | | |  |  |  |  |  |  |
| Tenosynovitis | 41 (75) | 23 (42) | 4.08 (1.81-9.15) | 0.001 | 3.36 (1.67-11.37) | 0.003 | 2.64 (1.07-6.48) | 0.034 |
| Synovitis | 25 (46) | 17 (31) | 1.86 (0.85-4.06) | 0.12 | 1.02 (0.38-2.75) | 0.96 |  |  |
| BME | 17 (31) | 22 (40) | 0.67 (0.31-1.47) | 0.32 | 0.55 (0.23-1.31) | 0.18 |  |  |
| Swollen joints, per joint | | | 1.57 (1.26-1.97) | <0.001 |  |  |  |  |
| Elevated CRP | |  | 3.23 (1.36-7.72) | 0.008 |  |  |  |  |

110 patients with undifferentiated arthritis (UA) were selected who were negative for both rheumatoid factor (RF) and anti-citrullinated protein antibody (ACPA). ¹On patient level a score was considered present or abnormal for BME, synovitis or tenosynovitis if, according to both readers, the score was present in <5% at the same location of symptom-free controls of the same age category. ²Multivariable analyses was performed for the foot and for the hand separately. SJC: swollen joint count (66 swollen joint counts were performed); CRP: C-reactive protein; OR: odds ratio; CI: confidence interval; MTP: metatarsophalangeal; MCP: metacarpophalangeal joint; BME: bone marrow edema.

# Table S4: Results of logistic regression for RA development in undifferentiated arthritis for the extensor and flexor tendons separately

|  | **Patients with MRI-feature (%)** | | **Univariable analyses** | | **Multivariable analyses: adjusted for local BME and synovitis²** | | **Multivariable analysis: adjusted for SJC and CRP²** | |
| --- | --- | --- | --- | --- | --- | --- | --- | --- |
|  | RA | no-RA | OR (95% CI) | P-value | OR (95% CI) | P-value | OR (95% CI) | P-value |
|  |  |  |  |  |  |  |  |  |
| **MTP 1-5¹** |  |  |  |  |  |  |  |  |
| Extensor tendon | 9 (14) | 5 (9) | 1.80 (0.57-5.72) | 0.32 | 2.03 (0.56-7.30) | 0.28 | 1.60 (0.45-5.74) | 0.47 |
| Flexor tendon | 14 (22) | 5 (9) | 3.09 (1.04-9.20) | 0.043 | 4.14 (1.03-16.68) | 0.046 | 2.11 (0.64-7.01) | 0.22 |
| **MCP 2-5¹** |  |  |  |  |  |  |  |  |
| Extensor tendon | 13 (20) | 5 (9) | 2.75 (0.92-8.27) | 0.071 | 3.21 (0.84-12.34) | 0.090 | 2.00 (0.57-7.00) | 0.28 |
| Flexor tendon | 26 (41) | 9 (15) | 3.80 (1.60-9.05) | 0.003 | 3.17 (1.29-7.78) | 0.012 | 2.54 (1.00-6.50) | 0.051 |

¹At joint level a score was considered abnormal for BME, synovitis or tenosynovitis if the scores of both readers at the same location was present in <5% of symptom-free persons of the same age category (18-40, 40-60, or >60 years). At patient level, BME, synovitis, tenosynovitis were considered present if ≥1 joint of foot or hand respectively was positive. ²Multivariable analyses was performed for the MTPs and MCPs separately. MRI: magnetic resonance imaging; RA: rheumatoid arthritis; BME: bone marrow edema; SJC: swollen joint count (66 swollen joint counts were performed); CRP: C-reactive protein; OR: odds ratio; CI: confidence interval; MTP: metatarsophalangeal; MCP: metacarpophalangeal joint.

# Table S5: Results of logistic regression for RA development in undifferentiated arthritis, for MRI-features at metacarpophalangeal and wrist joint separately

|  | **Patients with MRI-feature, n(%)** | | **Univariable analyses** | | **Multivariable analyses: presence of local BME, synovitis and tenosynovitis²** | | **Multivariable analysis: presence of tenosynovitis adjusted for SJC and CRP²** | |
| --- | --- | --- | --- | --- | --- | --- | --- | --- |
|  | RA | no-RA | OR (95% CI) | P-value | OR (95% CI) | P-value | OR (95% CI) | P-value |
|  |  |  |  |  |  |  |  |  |
| **MCPs**¹ |  |  |  |  |  |  |  |  |
| Tenosynovitis | 34 (53) | 14 (24) | 3.64 (1.68-7.91) | 0.001 | 3.61 (1.50-8.65) | 0.004 | 2.64 (1.14-6.16) | 0.024 |
| Synovitis | 22 (34) | 12 (20) | 2.05 (0.91-4.65) | 0.085 | 1.61 (0.58-4.47) | 0.36 |  |  |
| BME | 9 (14) | 13 (22) | 0.58 (0.23-1.48) | 0.25 | 0.34 (0.11-1.03) | 0.056 |  |  |
| **Wrist**¹ |  |  |  |  |  |  |  |  |
| Tenosynovitis | 36 (56) | 19 (32) | 2.71 (1.30-5.65) | 0.008 | 2.60 (1.19-5.69) | 0.017 | 1.45 (0.62-3.35) | 0.39 |
| Synovitis | 14 (22) | 8 (14) | 1.79 (0.69-4.63) | 0.23 | 1.27 (0.42-3.86) | 0.68 |  |  |
| BME | 17 (27) | 15 (25) | 1.06 (0.47-2.38) | 0.89 | 0.84 (0.34-2.09) | 0.71 |  |  |

¹ At joint level a score was considered abnormal for BME, synovitis or tenosynovitis if the scores of both readers at the same location was present in <5% of symptom-free persons of the same age category (18-40, 40-60, or >60 years). At patient level, BME, synovitis, tenosynovitis were considered present if ≥1 joint of foot or hand respectively was positive. ²Multivariable analyses was performed for the MCPs and wrist separately. OR: odds ratio; CI: confidence interval; MCP: metacarpophalangeal joint 2-5; BME: bone marrow edema.

# Figure S1: Frequencies of concomitant presence of MRI-detected inflammation for the foot and hand


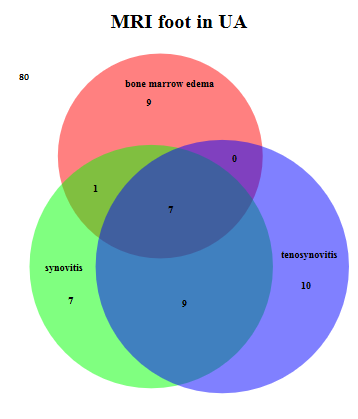


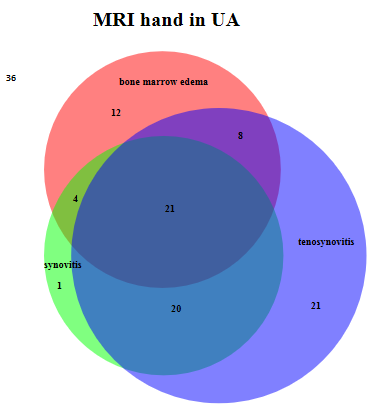


The foot is defined as MTP 1-5, the hand is defined as MCP 2-5 or the wrist. UA: undifferentiated arthritis.

# REFERENCES

1. Mayerhoefer ME, Breitenseher MJ, Kramer J, Aigner N, Norden C, Hofmann S. STIR vs. T1-weighted fat-suppressed gadolinium-enhanced MRI of bone marrow edema of the knee: computer-assisted quantitative comparison and influence of injected contrast media volume and acquisition parameters. J Magn Reson Imaging. 2005;22(6):788-93.

2. Schmid MR, Hodler J, Vienne P, Binkert CA, Zanetti M. Bone marrow abnormalities of foot and ankle: STIR versus T1-weighted contrast-enhanced fat-suppressed spin-echo MR imaging. Radiology. 2002;224(2):463-9.

3. Stomp W, Krabben A, van der Heijde D, Huizinga TW, Bloem JL, van der Helm-van Mil AH, et al. Aiming for a shorter rheumatoid arthritis MRI protocol: can contrast-enhanced MRI replace T2 for the detection of bone marrow oedema? Eur Radiol. 2014;24(10):2614-22.

4. Sudol-Szopinska I, Jurik AG, Eshed I, Lennart J, Grainger A, Ostergaard M, et al. Recommendations of the ESSR Arthritis Subcommittee for the Use of Magnetic Resonance Imaging in Musculoskeletal Rheumatic Diseases. Semin Musculoskelet Radiol. 2015;19(4):396-411.

5. Ostergaard M, Edmonds J, McQueen F, Peterfy C, Lassere M, Ejbjerg B, et al. An introduction to the EULAR-OMERACT rheumatoid arthritis MRI reference image atlas. Ann Rheum Dis. 2005;64 Suppl 1:i3-7.

6. Haavardsholm EA, Ostergaard M, Ejbjerg BJ, Kvan NP, Kvien TK. Introduction of a novel magnetic resonance imaging tenosynovitis score for rheumatoid arthritis: reliability in a multireader longitudinal study. Ann Rheum Dis. 2007;66(9):1216-20.
